# Independent prognostic value of fascin immunoreactivity in stage III–IV colonic adenocarcinoma

**DOI:** 10.1038/sj.bjc.6603690

**Published:** 2007-03-20

**Authors:** G Puppa, P Maisonneuve, A Sonzogni, M Masullo, A Chiappa, M Valerio, M G Zampino, I Franceschetti, P Capelli, M Chilosi, F Menestrina, G Viale, G Pelosi

**Affiliations:** 1 Division of Pathology, CRO-National Cancer Institute, Aviano, Italy; 2 Division of Epidemiology and Biostatistics, European Institute of Oncology, Via G. Ripamonti, Milano 435 I-20141, Italy; 3 Division of Pathology and Laboratory Medicine, European Institute of Oncology, Via G. Ripamonti, Milano 435 I-20141, Italy; 4 Division of General Surgery, European Institute of Oncology, Via G. Ripamonti, Milano 435 I-20141, Italy; 5 Division of Medical Oncology, European Institute of Oncology, Via G. Ripamonti, Milano 435 I-20141, Italy; 6 Institute of Pathology, University of Verona, Istituti Biologici, Strada Le Grazie 8-3714, Verona 37134, Italy; 7 University of Milan School of Medicine, Milan, Italy

**Keywords:** fascin, immunohistochemistry, colorectal cancer, prognosis

## Abstract

Fascin, an actin-bundling protein involved in cell motility, has been shown to be upregulated in several types of carcinomas. In this study, we investigated the expression of fascin in 228 advanced colonic adenocarcinoma patients with a long follow-up. Fascin expression was compared with several clinicopathologic parameters and survival. Overall, fascin immunoreactivity was detected in 162 (71%) tumours with a prevalence for right-sided tumours (*P*<0.001). Fascin correlated significantly with sex, tumour grade and stage, mucinous differentiation, number of metastatic lymph nodes, extranodal tumour extension, and the occurrence of distant metastases. Patients with fascin-expressing tumours experienced a shorter disease-free and overall survival in comparison with those with negative tumours, and fascin immunoreactivity emerged as an independent prognostic factor in the multivariate analysis. Moreover, patients with the same tumour stages could be stratified in different risk categories for relapse and progression according to fascin expression. Our findings suggest that fascin is a useful prognostic marker for colonic adenocarcinomas.

Colorectal cancer is among the most commonly diagnosed malignancies, ranking fourth in frequency in men and third in women (Parkin *et al*, 2005). Although the prognosis has slightly improved in the last few years (survival estimates at 5 years are now 65% in North America and 54% in Western Europe) (Parkin *et al*, 2005), colonic cancer is still a major cause of death worldwide.

The recent introduction of new chemotherapeutic combinations of adjuvant chemotherapy has improved the disease-free survival (DFS) of stage III patients (Gramont, 2005). Nevertheless, a consistent fraction of patients with locally advanced colonic carcinoma will relapse (Obrand and Gordon, 1997; Gramont, 2005), mostly within the first 3 years (Sadahiro *et al*, 2003). One of the reasons accounting for this failure may be the difficulty in stratifying patient groups with locally advanced disease in different risk categories. The issue of prognostic heterogeneity for patients with stage III colorectal cancer (Merkel *et al*, 2001) has, in part, been addressed by the last TNM edition, where colonic carcinoma patients are substaged according to the depth of the intestinal wall invasion and the number of lymph nodes involved (Greene *et al*, 2002). In this scenario, a large number of biomolecular markers have been suggested as new prognostic factors in locally advanced colonic carcinomas, but there are insufficient data or a poor clinical translation for most of them (Duffy *et al*, 2003; Funaioli *et al*, 2006).

The interaction among cancer cells, extracellular matrix proteins, and endothelial cells plays a major role in local invasion and distant metastases (Aznavoorian *et al*, 1993; Alessandro *et al*, 1996; Liotta and Kohn, 2001). Invasive tumour cells are often characterised by changes in cell shape with the appearance of membrane protrusions, loss of anchorage dependency, and loss of cell–cell adhesion and junctional communications. Many of these changes are related to re-arrangements of the cytoskeletal microfilaments because of the action of several types of actin cross-linking proteins (Maekawa *et al*, 1988; Tilney *et al*, 1998).

Among these molecules, fascins are involved in the organisation of two major forms of actin-based structures that include cortical cell protrusions such as filopodia, spikes, lamellipodial ribs, dendrites and microvilli, and cytoplasmic microfilament bundles (Kureishy *et al*, 2002). Fascin, which is expressed in normal mesenchymal, endothelial, dendritic, and neuronal cells but not in normal epithelia (Kureishy *et al*, 2002) is important in cell–cell interactions and adhesion (Adams *et al*, 2001), both interacting with specific tight (Wong *et al*, 1999) and adherens junctions (Tao *et al*, 1996) and intervening in focal adhesions via *β*1-integrin (Jawhari *et al*, 2003). Moreover, the protein promotes cell locomotion (Yamashiro *et al*, 1998) and participates in the mechanical organisation of stress fibres (Adams, 1997). A definite overexpression of fascin has been reported in carcinomas of different organs, including oesophagus (Hashimoto *et al*, 2005a; Xie *et al*, 2005; Zhang *et al*, 2006), stomach (Hashimoto *et al*, 2004), colon (Jawhari *et al*, 2003; Hashimoto *et al*, 2006), pancreas (Maitra *et al*, 2002), breast (Grothey *et al*, 2000; Yoder *et al*, 2005), skin (Goncharuk *et al*, 2002), lung (Pelosi *et al*, 2003a, 2003b; Choi *et al*, 2006), urinary bladder (Tong *et al*, 2005), and ovary (Hu *et al*, 2000) and the protein has been recently proposed as a novel biomarker for aggressive tumour behaviour (Hashimoto *et al*, 2005b, 2006; Zhang *et al*, 2006). In colonic adenocarcinoma cell lines, fascin overexpression correlates with an increase in the formation of dynamic cell protrusions, proliferation, and invasiveness (Jawhari *et al*, 2003). Besides, more recently, in CRC, fascin overexpression has been shown to correlate with proximal colon tumour location and shorter patients' survival (Hashimoto *et al*, 2006).

In this study, we investigated the prevalence and clinicopathological implications, including survival, of fascin immunoreactivity in either primary tumours or lymph node metastases of 228 patients with stage III–IV colonic adenocarcinoma. Our results support the view that fascin correlates with a tumour cell invasive phenotype, emerging as an independent predictor of reduced survival.

## MATERIALS AND METHODS

### Patients and tumours

Three hundred consecutive patients with stage III and IV (6th TNM classification of malignant tumours) advanced colorectal adenocarcinoma (231 colonic and 69 rectal) were identified and surgically treated at the European Institute of Oncology and at the University Hospital of Verona between 1988 and 1999. As this study was aimed at assessing the role of fascin in tumour progression of advanced colonic adenocarcinoma, we also included stage IV patients in the survival analysis, whereas the earlier stages of disease were not taken into account. Other inclusion criteria were no neo-adjuvant treatment and age ⩽85 years (three patients aged 87, 88, and 92 years were excluded). All the data were retrieved from the original clinical charts, databases of the participating institutions, through direct interviews with surviving patients or their family members, family physicians, or the death registry office. The data that was analysed included the date of birth, date of surgery, date of last follow-up/death, date of local recurrence, date of first metastatic spreading, site of metastatic spreading (classified as local recurrence, hepatic, abdominal extra-hepatic, and distant), and the anatomical location of the neoplasm (classified as right, transverse, and left colon).

Information on overall survival (OS) was available for all patients, with 141 of them (61.8%) dying from their disease. The mean survival period was 54.2 months (standard deviation 43.1; range 0–106 months). Recurrent disease information was assessable in 220 (96%) patients, with a mean time to event of 39.0 months (standard deviation 38.6; range 0–170 months). Information on adjuvant therapy was available for 205 patients (90%) (116 treated and 89 not treated). Because of the different biological, clinicopathologic, and therapeutic implications of rectal cancer (with particular reference to the role of additional therapies in the management of these patients) (Rodel and Sauer, 2004), rectal adenocarcinomas were recorded for the prevalence of fascin immunoreactivity but then excluded from any further clinicopathologic and survival analysis.

The paraffin blocks of all cases were retrieved and the original haematoxylin and eosin-stained sections reviewed for confirmation of pT3 or pT4 status, histologic tumour grade (categorised with a three-tier grading system using the extent of gland formation and microtubular structures as the primary grade criteria (Grinnell, 1939; Jass *et al*, 1986), tumour type, occurrence of vascular (classified as intramural and extramural venous vessels or lymphatic channels), and perineural invasion; tumour growth patterns (expansive or infiltrative margins); number of metastatic lymph nodes, and the occurrence of extracapsular lymph node involvement.

### Immunocytochemistry and evaluation of data

Formalin-fixed and paraffin-embedded tissue samples obtained at surgery were investigated. Tumours up to 2 cm in size were entirely embedded and immunostained. Ten samples of normal colonic wall from patients with nonmalignant intestinal diseases and peritumoural colonic tissue from the same cohort of patients investigated provided the control groups for noncarcinoma and carcinoma patients, respectively.

Fascin was immunolocalised on 4-*μ*m-thin tissue sections using the monoclonal antibody 55k-2 (Dako, Glostrup, Denmark) recognising a 55-kDa protein in Western blots of HeLa, normal rat kidney, and gerbil fibroma cell lysates (Yamashiro-Matsumura and Matsumura, 1986). This anti-fascin antibody was also found to immunoblot bacterially expressed *human homolog singed gene* (hsn) product and a 55-kDa protein from cell lysates of peripheral blood dendritic cells (Duh *et al*, 1994; Mosialos *et al*, 1996). After blockage of endogenous peroxidase activity with 5% hydrogen peroxide and heat-mediated antigen retrieval with 1 mM EDTA, pH 8 (Sigma Chemical Co, St Louis, MO, USA) in a microwave oven at 750 W for 12 min, the sections were reacted overnight at 4°C with the primary antibody at a dilution of 2.5 *μ*g ml^−1^ in Tris-buffered saline. Detection was performed using a commercially available kit (Dako EnVision Plus-HRP, Dako), according to the manufacturer's instructions. Peroxidase activity was developed with 3-3′-diaminobenzidine-copper sulphate (Sigma Chemical Co) to obtain a brownish-black end product. Tumour proliferative fraction was assessed by Ki-67 immunostaining and the percentage of reactive cells was recorded as the labelling index according to previously refined immunohistochemical method (Pelosi *et al*, 1996). The specificity of all immunoreactions was double-checked by substituting the primary antibodies with nonrelated isotypic mouse immunoglobulins at a comparable dilution and with normal mouse serum alone (Burry, 2000).

Two observers (GP and GP) evaluated fascin immunoreactivity of tumour cells independently and blindly, without knowledge of the patients' clinical outcome or of the tumour features. Results were expressed according to the percentage of immunoreactive cells among at least 2000 tumour cells in consecutive unselected histological fields. Intensity of fascin immunostaining was also graded as low (expressed as ‘1’), if a faint to distinct granular staining was detectable throughout the cytoplasm but less intense than seen in the normal internal controls (endothelial and dendritic cells), or strong (expressed as ‘2’), if it was of the same or greater intensity than the control staining. A score was then calculated multiplying the percentage of immunoreactive cells by the intensity level of immunostaining. Therefore, tumours were considered negative if staining was completely absent in the neoplastic cells; moderately positive if the fascin score ranged from 1 to 100, and strongly positive if it was higher than 100.

### Statistical analysis

Associations between intramural and extramural fascin expression and clinicopathological characteristics of the tumour were assessed by the Fisher's exact test for categorical variables and with the Mantel–Haenszel *χ*^2^ test for trend for ordinal variables. Disease-free survival was calculated from the date of surgery until the date of either loco-regional relapse or the development of distant metastasis. Only mortality from disease was considered for OS analysis. Disease-free and OS curves were obtained using the Kaplan–Meier method, whereas the comparison between the groups was assessed by the log-rank test, considering a *P*-value <0.05 as significant. The univariate and multivariate Cox proportional hazard regression was used to assess the prognostic significance of fascin and other clinical characteristics on survival. As this study dealt with locally advanced and metastatic tumours that actually have a poor prognosis, patients were censored at 6 years to emphasize the prognostic role of fascin expression as an early predictive marker for tumour progression. All tests were two-sided. Analyses were performed with the SAS software (Cary, NC, USA).

## RESULTS

### Patients

The study encompassed 121 men (median age 66 years, range 38–84) and 107 women (median age 65, range 30–84).

Stage distribution included nine (4%) tumour patients with stage IIIA (six men and three women), 100 (44%) with stage IIIB (49 men and 51 women), 61 (26%) with stage IIIC (29 men and 32 women), and 58 (25%) with stage IV (37 men and 21 women). Eighty-five patients (37%) had right colon carcinoma, 14 (6%) transverse colon carcinoma, and 129 (57%) left colon carcinoma. Twenty patients (9%) experienced local recurrences and 134 (59%) distant metastases, classified as hepatic (80 patients) abdominal extra-hepatic (22 patients), and extra-abdominal (32 patients). Sixteen (7%) of the adenocarcinomas were grade 1, 144 (63%) grade 2, and 68 (30%) grade 3, with 213 (93%) of them being typical adenocarcinoma, 13 (6%) mucinous and two (1%) signet ring cell adenocarcinoma. Intramural venous invasion was detected in 51 (22%) cases, extramural venous invasion in 69 (30%), lymphatic vessel invasion in 21 (9%), and perineural invasion in 29 (13%). The distribution of these morphological features was independent of the tumour anatomical location, except for the tumour grade and the presence of intramural venous invasion, with a net prevalence of high-grade tumours and intramural vascular invasion in the right colon as compared with the left (*P*<0.001). The survival curves of patients treated in the two participating centres were not statistically different.

### Immunohistochemical data

The normal colonic epithelium consistently lacked any fascin immunoreactivity, which was instead encountered in the underlying lamina propria and tumour stroma, with the endothelial and fibroblastic cells showing a consistent and strong immunoreactivity for fascin.

**Figure 1:**
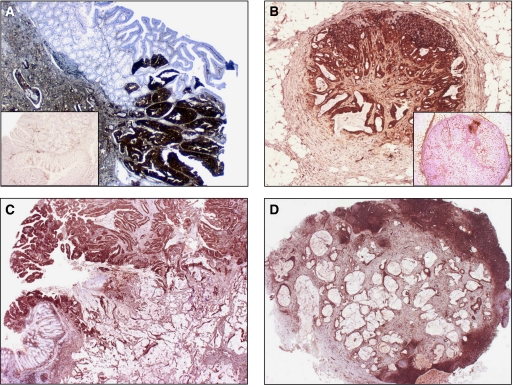
(**A**–**D**) Fascin expression in primary and metastatic lymph nodes, in conventional (**A** and **B**) and mucinous adenocarcinoma (**C** and **D**), both showing fascin score >100. Two other examples of adenocarcinoma (**A**) and lymph node metastasis (**B**) lacking any fascin immunoreactivity are also shown (insets).

**Figure 2:**
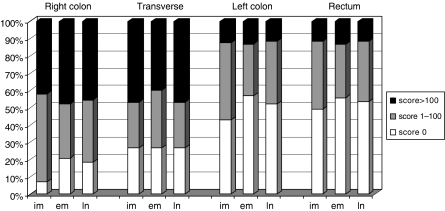
The distribution of the fascin score along the large bowel according to the intramural (im), extramural (em), and lymph node (ln) compartments is shown. A differential distribution was noted among the diverse intestinal tracts (*P*<0.001), with a prevalence of positive fascin score in the intramural component of the right colon only as compared with the extramural or lymph node location (*P*=0.025).

Representative examples of fascin immunoreactivity are depicted in Figure 1, and its distribution along the large bowel according to intramural, extramural, and lymph node location is reported in Figure 2. A positive fascin score was detected in the intramural component of 162 (71%) specimens (intramural fascin), in the extramural component of 132 (58%) (extramural fascin), and in metastatic lymph nodes of 141 (62%) patients.

Thirty-five tumours showed a positive fascin score in the intramural but not in the extramural component; the opposite was seen in only four tumours.

Among the 162 tumours positives in the intramural component, 130 had positive lymph node metastasis (80%) and among the 132 tumours positives in the extramural component 116 had positive lymph node metastasis (88%).

Striking differences in the prevalence of fascin immunoreactivity between right- and left-sided colon segments were found (Figure 2 and Table 1).

An intratumoural, extratumoural, and lymph node fascin score >100 was significantly associated with the female sex, tumour location in the right or transverse colon, more advanced tumour stage, pN class, the pattern of lymph node involvement (with a higher level of immunoreactivity in lymph nodes showing extracapsular extension), higher tumour grade, mucinous histotype, and the occurrence of metastasis and of a heavy peritumoural lymphoid infiltrate (Table 1). No correlations were found with the other variables, including tumour proliferative fraction, chemotherapeutic regimens (data not shown), and site of metastasis.

**Figure 3:**
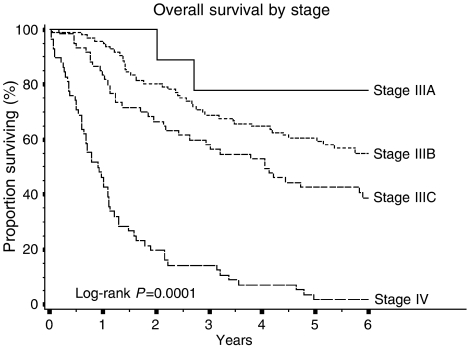
Overall survival analysis according to tumour stage in 228 colon adenocarcinoma patients.

Regarding survival, traditional prognostic variables such as the pT, pN, tumour grade, and vascular invasion correlated well with the patients' life expectation (Table 2), with tumour stage according to the TNM system being a strong predictor of survival (Figure 3). A correlation with poorer clinical outcome was also noted for mucinous differentiation and right tumour location (Table 2).

**Figure 4:**
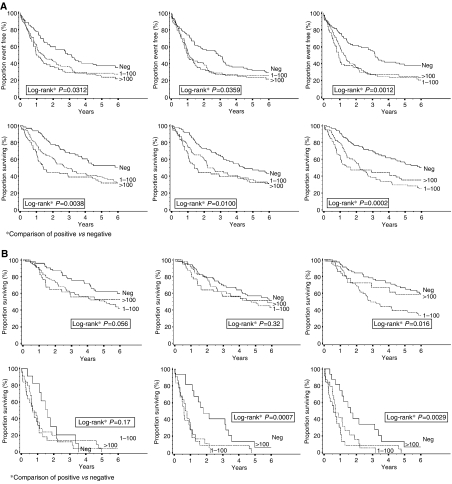
(**A**) Disease-free and OS analysis of 228 colon adenocarcinoma patients according to intramural, extramural, and lymph node fascin expression. (**B**) Overall survival of patients with stage III (top) and IV (bottom) colon adenocarcinoma according to intramural, extramural, and lymph node fascin expression.

A positive fascin score (independent of its threshold) at any site was significantly associated with both a shorter OS and DFS (Figure 4A and B), even for patients with stage IV disease (Figure 4B, bottom). In the multivariate analysis, fascin emerged as an independent prognostic factor for both OS and DFS, together with stage and a mucinous-signet ring histotype (Table 3).

## DISCUSSION

In this study, we investigated a large series of colorectal carcinomas for fascin immunoreactivity. The main results are that fascin is upregulated in most colonic carcinomas, correlating with a higher tumour grade, right tumour location and tumour stage, and that fascin immunoreactivity is an independent predictor of reduced OS and DFS in patients with advanced tumour stage.

A growing body of literature reports that fascin is expressed in many types of transformed epithelial cell lines and in several solid neoplasms (Grothey *et al*, 2000; Hu *et al*, 2000; Goncharuk *et al*, 2002; Jawhari *et al*, 2003; Pelosi *et al*, 2003a, 2003b; Hashimoto *et al*, 2004, 2005a, 2006; Tong *et al*, 2005; Xie *et al*, 2005; Yoder *et al*, 2005; Choi *et al*, 2006; Zhang *et al*, 2006) where it correlates with tumour stage (Hashimoto *et al*, 2004, 2005a; Yoder *et al*, 2005; Choi *et al*, 2006) and grade (Pelosi *et al*, 2003b; Hashimoto *et al*, 2004; Yoder *et al*, 2005), pT class (Hashimoto *et al*, 2004, 2005a), lymph node involvement (Hashimoto *et al*, 2004, 2005a; Choi *et al*, 2006; Zhang *et al*, 2006), recurrence (Hashimoto *et al*, 2004), and both OS (Hashimoto *et al*, 2004, 2005a, 2006; Yoder *et al*, 2005) and DFS (Pelosi *et al*, 2003b; Yoder *et al*, 2005). The close association we found between fascin immunoreactivity and tumour stage, tumour grade, the number and type of lymph node involvement, and distant metastasis indicates the major role of fascin in the progression of colonic adenocarcinoma. In our study, fascin seems to play a different role in the progression of right- and tranverse-sided neoplasms (as recently evidenciated) (Hashimoto *et al*, 2006) and of mucinous variants of colonic adenocarcinomas as compared with the other types of colonic cancers (Table 2). Pelosi *et al* (2003b) also noted a differential distribution of fascin immunoreactivity in mucinous and nonmucinous bronchioloalveolar carcinomas of the lung and fascin transfection in colonic carcinoma cell lines increased the levels of neutral mucin (Jawhari *et al*, 2003). Although the clinical implications of colorectal mucinous carcinoma are still controversial, a consensus conference on colorectal carcinomas stated that mucinous and signet ring carcinomas, when evaluated jointly, have a poorer prognosis than intestinal-type adenocarcinomas (Compton *et al*, 2000).

Although the association of fascin expression with lymph node involvement has already been documented for other gastrointestinal cancers such as oesophageal and gastric carcinomas (Hashimoto *et al*, 2004, 2005a) and also for pulmonary neoplasms (Pelosi *et al*, 2003a; Choi *et al*, 2006), we have shown that fascin immunoreactivity in colonic adenocarcinomas is also related to the patterns of lymph node involvement (intra- *vs* extra-lymph nodal colonisation). As fascin immunoreactivity is also associated with the number of metastatic lymph nodes and the occurrence of distant metastases, we hypothesise that this molecule is very likely involved in the metastatic process of colonic adenocarcinoma cells via its motility-inducing properties. This is further sustained by the observation that patients with lymph node metastases immunoreactive for fascin experienced a more aggressive clinical course than patients with negative lymph node metastases (Figure 4A). This significance was kept stratifying the prognostic impact of fascin according to tumour stages III and IV (Figure 4B). Thus, fascin expression in primary and metastatic tumours could unveil the different clinical aggressiveness of tumours that are otherwise classified in the same risk category.

Fascin immunoreactivity was not associated with the tumour proliferation fraction as assessed by the Ki-67-labelling index, at variance with previous studies of lung and stomach cancers and also of colonic cell cultures (Jawhari *et al*, 2003; Pelosi *et al*, 2003b; Hashimoto *et al*, 2004). In a recent work on CRC, similar to the current one, comparing fascin expression with Ki-67 immunostaining, a lack of direct association between the two markers was noted, indicating that the fascin upregulation do not correlate positively with cell proliferation (Hashimoto *et al*, 2006).

However, the prognostic role of the Ki-67-labelling index in colorectal carcinoma is still highly controversial and probably different from other solid neoplasms. In fact, recent observations document that a high Ki-67-labelling index is associated with better OS (Allegra *et al*, 2003) in both treated and untreated patients (Garrity *et al*, 2004), as reported in the current series (Table 2).

Fascin immunoreactivity was associated with a shorter OS and DFS, independent of tumour stage, which is the most important prognostic factor in this tumour type (Compton and Greene, 2004). In the multivariate analysis, similar results were also obtained for other tumour types, such as pulmonary (Pelosi *et al*, 2003a), oesophageal (Hashimoto *et al*, 2005a), breast (Yoder *et al*, 2005) carcinomas and more recently CRC (Hashimoto *et al*, 2006). Our findings confirming that fascin is a negative prognostic factor for advanced colonic adenocarcinoma encourage clinical translation, especially when considering that the current substaging of colorectal cancer according to the latest TNM classification emphasizes the prognostic heterogeneity of patients within the same tumour stage group. The different prognostic implications of lymph node metastases according to the amount of fascin could well be incorporated in new staging proposals. Finally, the identification of patients with a reduced life expectation according to the degree of fascin expressed by their respective tumours also justifies the potential use of novel targeted therapies, as recently proposed for other malignant epithelial neoplasms (Hashimoto *et al*, 2005b, 2006).

Additional studies are needed to investigate the role of fascin in right-sided colonic cancer and in mucinous differentiation.

## Figures and Tables

**Table 1:** Association between intramural and extramural fascin expression and other clinicopathological characteristics in colon cancer

	**Intramural fascin score**				**Extramural fascin score**				**Lymph nodal fascin score**			
	**Neg**	**⩽100**	**>100**	***P*-value**	**Neg**	**⩽100**	**>100**	***P*-value**	**Neg**	**⩽100**	**>100**	***P*-value**
*Sex*												
Male	43	54	24		50	41	24		51	46	23	
Female	23	51	33	0.010	35	29	38	0.022	36	34	37	0.022
												
*Age*												
<50	10	12	3		13	5	5		14	6	5	
50–59	9	29	11		13	22	12		13	22	14	
60–69	23	31	13		27	20	15		29	21	17	
70+	24	33	30	0.120^a^	32	23	30	0.247^a^	31	31	24	0.489^a^
												
*Localisation*												
Right	6	44	35		16	27	40		16	31	38	
Transverse	4	4	6		4	5	5		4	2	8	
Left	56	57	16	<0.0001	65	38	17	<0.0001	67	47	14	<0.0001
												
*Histology*												
Nonmucinous	65	98	50		83	67	53		86	74	52	
Mucinous	1	7	7	0.017	2	3	9	0.0042	1	6	8	0.0033
												
*Grade*												
G1	5	10	1		8	5	3		9	4	3	
G2	51	65	28		64	42	30		62	55	26	
G3	10	30	28	<0.0001^a^	13	23	29	<0.0001^a^	16	21	31	<0.0001^a^
												
*pT*												
pT1	0	1	1		0	1	1		1	0	0	
pT2	3	8	1		0	1	0		5	7	0	
pT3	56	85	44		76	60	49		72	64	49	
pT4	7	11	11	0.38^a^	9	8	12	0.47^a^	9	9	11	0.042^a^
												
*pN*												
N1	42	69	28		55	44	30		55	57	26	
N2	24	36	29	0.12	30	26	32	0.056	32	23	34	0.032
												
*Number of positive nodes*												
1	18	33	11		25	20	11		25	24	12	
2	19	23	10		22	15	12		22	22	8	
3–5	23	28	18		30	22	16		29	21	19	
>5	6	21	18	0.014^a^	8	13	23	0.0018^a^	11	13	21	0.0079^a^
												
*LN met*												
Intra LN	41	65	23		55	41	26		54	51	23	
Extra LN	25	40	34	0.019	30	29	36	0.0072	33	29	37	0.0081
												
*pM*												
M0	55	80	35		69	52	40		71	61	37	
M1	11	25	22	0.0060	16	18	22	0.024	16	19	23	0.0080
												
*Stage*												
IIIA	3	5	1		0	2	1		4	4	1	
IIIB	30	50	20		44	31	23		40	42	17	
IIIC	22	25	14		25	19	16		27	15	19	
IV	11	25	22	0.021^a^	16	18	22	0.051^a^	16	19	23	0.0064^a^
												
*Ki-67 intramural*												
<40	18	26	15		20	20	16		19	28	12	
40–59	21	31	12		28	19	15		28	22	14	
60+	27	46	30	0.40^a^	36	30	31	0.75^a^	39	29	34	0.48^a^
												
*Ki-67 extramural*												
<40	26	33	16		28	27	20		27	32	16	
40–59	14	21	16		20	17	14		19	16	15	
60+	23	42	24	0.23^a^	36	25	28	0.89^a^	35	25	29	0.54^a^

LN, lymph node.

No association was found between fascin expression and centre, chemotherapeutic treatment, vascular, lymphatic, and neural invasion.

a *P*-value for trend using the Mantel–Haenszel *χ*^2^ test.

**Table 2:** Univariate analysis for disease-free and overall survival

	**Disease free survival**		**Overall survival (death from cancer)**	
**Univariate analysis**	**HR (95% CI)**	***P*-value**	**HR (95% CI)**	***P*-value**
*Fascin intramural*				
Negative	1.00		1.00	
⩽100	1.38 (0.93–2.05)	0.11	**1.69 (1.11–2.59)**	**0.015**
>100	**1.64 (1.08–2.48)**	**0.020**	**2.01 (1.25–3.23)**	**0.004**
				
*Fascin intramural*				
Absent	1.00		1.00	
Present	**1.48 (1.03–2.12)**	**0.032**	**1.79 (1.20–2.68)**	**0.004**
				
*Fascin extramural*				
Negative	1.00		1.00	
⩽100	1.31 (0.89–1.95)	0.15	1.50 (1.00–2.26)	0.057
100+	**1.53 (1.05–2.23)**	**0.028**	**1.71 (1.13–2.60)**	**0.012**
				
*Fascin extramural*				
Absent	1.00		1.00	
Present	**1.42 (1.02–1.97)**	**0.037**	**1.59 (1.11–2.28)**	**0.011**
				
*Fascin LN*				
Negative	1.00		1.00	
⩽100	**1.69 (1.16–2.45)**	**0.0058**	**2.04 (1.37–3.05)**	**0.0005**
100+	**1.81 (1.20–2.71)**	**0.0045**	**1.87 (1.20–2.90)**	**0.0056**
				
*Fascin LN*				
Absent	1.00		1.00	
Present	**1.74 (1.24–2.43)**	**0.0013**	**1.97 (1.36–2.84)**	**0.0003**
				
*Localization*				
Left	1.00		1.00	
Right-transverse	**1.42 (1.04–1.95)**	**0.029**	**1.44 (1.03–2.01)**	**0.033**
				
*Histology*				
Nonmucinous	1.00		1.00	
Mucinous	1.66 (0.94–2.94)	0.083	**2.11 (1.19–3.75)**	**0.011**
				
*Grade*				
G1	1.00		1.00	
G2	2.05 (0.96–4.42)	0.083	2.44 (0.97–6.01)	0.052
G3	**2.43 (1.10–5.37)**	**0.029**	**2.96 (1.17–7.49)**	**0.022**
				
*PT*				
pT1–2	1.00		1.00	
pT3–4	**4.01 (1.48–10.8)**	**0.0062**	**2.93 (1.08–7.92)**	**0.034**
				
*PN*				
N1	1.00		1.00	
N2	**1.74 (1.27–2.39)**	**0.0006**	**1.56 (1.11–2.19)**	**0.0096**
				
*Number of positive nodes*				
1	1.00		1.00	
2–5	**1.65 (1.10–2.45)**	**0.015**	**1.59 (1.03–2.46)**	**0.035**
>5	**2.91 (1.82–4.63)**	**<0.00001**	**3.30 (2.03–5.37)**	**<0.0001**
				
*LN met*				
Intra LN	1.00		1.00	
Extra LN	**1.79 (1.31–2.45)**	**0.0003**	**1.79 (1.28–2.50)**	**0.0007**
				
*PM*				
M0	1.00		1.00	
M1	**3.67 (2.62–5.15)**	**<0.0001**	**5.93 (4.13–8.52)**	**<0.0001**
				
*Stage*				
IIIA	0.30 (0.07–1.12	0.091	0.43 (0.11–1.77)	0.24
IIIB	1.00		1.00	
IIIC	**1.75 (1.17–2.63)**	**0.0065**	**1.62 (1.04–2.52)**	**0.033**
IV	**4.31 (2.94–6.32)**	**<0.0001**	**6.92 (4.56–10.5)**	**<0.0001**
				
*Venous invasion*				
Absent	1.00		1.00	
Present	**1.64 (1.20–2.25)**	**0.0020**	**1.65 (1.18–2.31)**	**0.0035!**
*Intramural*				
Absent	1.00		1.00	
Present	1.34 (0.93–1.93)	0.12	**1.56 (1.07–2.29)**	**0.022**
				
*Extramural*				
Absent	1.00		1.00	
Present	**1.74 (1.25–2.42)**	**0.0010**	**1.63 (1.14–2.32)**	**0.0069**
				
*Ki-67 intramural* a				
<40	1.00		1.00	
40–59	0.84 (0.56–1.26)	0.39	0.72 (0.47–1.10)	0.13
60+	0.75 (0.51–1.11)	0.15	**0.60 (0.40–0.91)**	**0.015**
				
*Ki-67 extramural* a				
<40	1.00		1.00	
40–59	0.89 (0.58–1.35)	0.57	0.73 (0.47–1.13)	0.16
60+	0.79 (0.55–1.14)	0.20	**0.66 (0.45–0.98)**	**0.039**

CI, confidence interval; HR, hazards ratio; LN, lymph node.

No association was found between survival and center, sex, age, lymphatic invasion, neural invasion, tumour border, peritumoral inflammation, and adjuvant treatment.

a These variables were associated with better survival.

**Table 3:** Multivariate analysis^a^ for disease-free and overall survival

		**Intramural**	**Extramural**	**LN**	**At any site**
*Disease free survival*					
Fascin	Present *vs* absent	**1.52 (1.04–2.23)**	**1.54 (1.09–2.19)**	**1.72 (1.22–2.45)**	**1.61 (1.07–2.42)**
Histology	Mucinous *vs* other	1.38 (0.77–2.47)	1.32 (0.73–2.37)	1.27 (0.70–2.28)	1.39 (0.78–2.50)
Stage	IIIa *vs* IIIb	0.29 (0.07–1.20)	0.41 (0.09–1.84)	0.29 (0.07–1.19)	0.34 (0.08–1.41)
	IIIc *vs* IIIb	**1.94 (1.29–2.93)**	**1.84 (1.22–2.76)**	**1.78 (1.18–2.67)**	**1.95 (1.29–2.94)**
	IV *vs* IIIb	**4.63 (3.09–6.92)**	**4.78 (3.20–7.14)**	**4.51 (3.02–6.74)**	**4.74 (3.17–7.08)**
					
*Overall survival*					
Fascin	Present *vs* absent	**1.74 (1.14–2.65)**	**1.71 (1.18–2.48)**	**1.96 (1.35–2.86)**	**1.92 (1.21–3.03)**
Histology	Mucinous *vs* other	**1.80 (1.00–3.23)**	1.72 (0.95–3.10)	1.64 (0.91–2.95)	**1.81** (**1.01–3.25)**
Stage	IIIa *vs* IIIb	0.43 (0.10–1.79)	0.51 (0.11–2.47)	0.44 (0.11–1.81)	0.49 (0.12–2.05)
	IIIc *vs* IIIb	**1.83 (1.17–2.88)**	**1.70 (1.09–2.67)**	**1.69 (1.08–2.64)**	**1.86 (1.19–2.91)**
	IV *vs* IIIb	**7.47 (4.83–11.6)**	**7.92 (5.10–12.3)**	**7.59 (4.89–11.8)**	**7.73 (4.99–12.0)**

LN, lymph node.

Also adjusted for age and sex.

a Only variables that retains statistical significance were kept in the final model. Bold numbers indicate a *P*-value <0.05.

## References

[bib1] Adams JC (1997) Characterization of cell-matrix adhesion requirements for the formation of fascin microspikes. Mol Biol Cell 8: 2345–2363 9362073 10.1091/mbc.8.11.2345 PMC25712

[bib2] Adams JC, Kureishy N, Taylor AL (2001) A role for syndecan-1 in coupling fascin spike formation by thrombospondin-1. J Cell Biol 152: 1169–1182 11257118 10.1083/jcb.152.6.1169 PMC2199199

[bib3] Alessandro R, Masiero L, Liotta LA, Kohn EC (1996) The role of calcium in the regulation of invasion and angiogenesis. *In vivo* 10: 153–160 8744794

[bib4] Allegra CJ, Paik S, Colangelo LH, Parr AL, Kirsch I, Kim G, Klein P, Johnston PG, Wolmark N, Wieand HS (2003) Prognostic value of thymidylate synthase, Ki-67, and p53 in patients with Dukes' B and C colon cancer: a National Cancer Institute-National Surgical Adjuvant Breast and Bowel Project collaborative study. J Clin Oncol 21: 241–250 12525515 10.1200/JCO.2003.05.044

[bib5] Aznavoorian S, Murphy AN, Stetler-Stevenson WG, Liotta LA (1993) Molecular aspects of tumor cell invasion and metastasis. Cancer 71: 1368–1383 8435813 10.1002/1097-0142(19930215)71:4<1368::aid-cncr2820710432>3.0.co;2-l

[bib6] Burry RW (2000) Specificity controls for immunocytochemical methods. J Histochem Cytochem 48: 163–166 10639482 10.1177/002215540004800201

[bib7] Choi PJ, Yang DK, Son CH, Lee KE, Lee JI, Roh MS (2006) Fascin immunoreactivity for preoperatively predicting lymph node metastases in peripheral adenocarcinoma of the lung 3 cm or less in diameter. Eur J Cardiothorac Surg 30: 538–542 16870459 10.1016/j.ejcts.2006.06.029

[bib8] Compton C, Fenoglio-Preiser CM, Pettigrew N, Fielding LP (2000) American Joint Committee on Cancer Prognostic Factors Consensus Conference: Colorectal Working Group. Cancer 88: 1739–1757 10738234 10.1002/(sici)1097-0142(20000401)88:7<1739::aid-cncr30>3.0.co;2-t

[bib9] Compton CC, Greene FL (2004) The staging of colorectal cancer: 2004 and beyond. CA Cancer J Clin 54: 295–308 15537574 10.3322/canjclin.54.6.295

[bib10] Duffy MJ, van DA, Haglund C, Hansson L, Klapdor R, Lamerz R, Nilsson O, Sturgeon C, Topolcan O (2003) Clinical utility of biochemical markers in colorectal cancer: European Group on Tumour Markers (EGTM) guidelines. Eur J Cancer 39: 718–727 12651195 10.1016/s0959-8049(02)00811-0

[bib11] Duh FM, Latif F, Weng Y, Geil L, Modi W, Stackhouse T, Matsumura F, Duan DR, Linehan WM, Lerman MI (1994) cDNA cloning and expression of the human homolog of the sea urchin fascin and *Drosophila* singed genes which encodes an actin-bundling protein. DNA Cell Biol 13: 821–827 8068206 10.1089/dna.1994.13.821

[bib12] Funaioli C, Pinto C, Mutri V, Di FF, Ceccarelli C, Martoni AA (2006) Does biomolecular characterization of stage II/III colorectal cancer have any prognostic value? Clin Colorectal Cancer 6: 38–45 16796790 10.3816/ccc.2006.n.019

[bib13] Garrity MM, Burgart LJ, Mahoney MR, Windschitl HE, Salim M, Wiesenfeld M, Krook JE, Michalak JC, Goldberg RM, O'Connell MJ, Furth AF, Sargent DJ, Murphy LM, Hill E, Riehle DL, Meyers CH, Witzig TE (2004) Prognostic value of proliferation, apoptosis, defective DNA mismatch repair, and p53 overexpression in patients with resected Dukes' B2 or C colon cancer: a North Central Cancer Treatment Group Study. J Clin Oncol 22: 1572–1582 15117979 10.1200/JCO.2004.10.042

[bib14] Goncharuk VN, Ross JS, Carlson JA (2002) Actin-binding protein fascin expression in skin neoplasia. J Cutan Pathol 29: 430–438 12139639 10.1034/j.1600-0560.2002.290708.x

[bib15] Gramont A (2005) Adjuvant therapy of stage II and III colon cancer. Semin Oncol 32: 11–14 10.1053/j.seminoncol.2005.06.004 16360006

[bib16] Greene FL, Stewart AK, Norton HJ (2002) A new TNM staging strategy for node-positive (stage III) colon cancer: an analysis of 50 042 patients. Ann Surg 236: 416–421 12368669 10.1097/00000658-200210000-00003 PMC1422595

[bib17] Grinnell RS (1939) The grading and prognosis of carcinoma of the colon and rectum. Ann Surg 109: 500–533 17857341 10.1097/00000658-193904000-00002 PMC1391306

[bib18] Grothey A, Hashizume R, Sahin AA, McCrea PD (2000) Fascin, an actin-bundling protein associated with cell motility, is upregulated in hormone receptor negative breast cancer. Br J Cancer 83: 870–873 10970687 10.1054/bjoc.2000.1395 PMC2374674

[bib19] Hashimoto Y, Ito T, Inoue H, Okumura T, Tanaka E, Tsunoda S, Higashiyama M, Watanabe G, Imamura M, Shimada Y (2005a) Prognostic significance of fascin overexpression in human esophageal squamous cell carcinoma. Clin Cancer Res 11: 2597–2605 15814639 10.1158/1078-0432.CCR-04-1378

[bib20] Hashimoto Y, Shimada Y, Kawamura J, Yamasaki S, Imamura M (2004) The prognostic relevance of fascin expression in human gastric carcinoma. Oncology 67: 262–270 15557788 10.1159/000081327

[bib21] Hashimoto Y, Skacel M, Adams JC (2005b) Roles of fascin in human carcinoma motility and signaling: prospects for a novel biomarker? Int J Biochem Cell Biol 37: 1787–1804 16002322 10.1016/j.biocel.2005.05.004

[bib22] Hashimoto Y, Skacel M, Lavery IC, Mukherjee AL, Casey G, Adams JC (2006) Prognostic significance of fascin expression in advanced colorectal cancer: an immunohistochemical study of colorectal adenomas and adenocarcinomas. BMC Cancer 6: 241 17029629 10.1186/1471-2407-6-241 PMC1615879

[bib23] Hu W, McCrea PD, Deavers M, Kavanagh JJ, Kudelka AP, Verschraegen CF (2000) Increased expression of fascin, motility associated protein, in cell cultures derived from ovarian cancer and in borderline and carcinomatous ovarian tumors. Clin Exp Metastasis 18: 83–88 11206843 10.1023/a:1026596609969

[bib24] Jass JR, Atkin WS, Cuzick J, Bussey HJ, Morson BC, Northover JM, Todd IP (1986) The grading of rectal cancer: historical perspectives and a multivariate analysis of 447 cases. Histopathology 10: 437–459 3721406 10.1111/j.1365-2559.1986.tb02497.x

[bib25] Jawhari AU, Buda A, Jenkins M, Shehzad K, Sarraf C, Noda M, Farthing MJ, Pignatelli M, Adams JC (2003) Fascin, an actin-bundling protein, modulates colonic epithelial cell invasiveness and differentiation *in vitro*. Am J Pathol 162: 69–80 12507891 10.1016/S0002-9440(10)63799-6 PMC1851132

[bib26] Kureishy N, Sapountzi V, Prag S, Anilkumar N, Adams JC (2002) Fascins, and their roles in cell structure and function. Bioessays 24: 350–361 11948621 10.1002/bies.10070

[bib27] Liotta LA, Kohn EC (2001) The microenvironment of the tumour-host interface. Nature 411: 375–379 11357145 10.1038/35077241

[bib28] Maekawa S, Ohta K, Sakai H (1988) A novel 53 kDa actin binding protein from porcine brain – further biochemical and immunological characterization. Cell Struct Funct 13: 373–385 3224379 10.1247/csf.13.373

[bib29] Maitra A, Iacobuzio-Donahue C, Rahman A, Sohn TA, Argani P, Meyer R, Yeo CJ, Cameron JL, Goggins M, Kern SE, Ashfaq R, Hruban RH, Wilentz RE (2002) Immunohistochemical validation of a novel epithelial and a novel stromal marker of pancreatic ductal adenocarcinoma identified by global expression microarrays: sea urchin fascin homolog and heat shock protein 47. Am J Clin Pathol 118: 52–59 12109856 10.1309/3PAM-P5WL-2LV0-R4EG

[bib30] Merkel S, Mansmann U, Papadopoulos T, Wittekind C, Hohenberger W, Hermanek P (2001) The prognostic inhomogeneity of colorectal carcinomas Stage III: a proposal for subdivision of Stage III. Cancer 92: 2754–2759 11753948

[bib31] Mosialos G, Birkenbach M, Ayehunie S, Matsumura F, Pinkus GS, Kieff E, Langhoff E (1996) Circulating human dendritic cells differentially express high levels of a 55-kd actin-bundling protein. Am J Pathol 148: 593–600 8579121 PMC1861678

[bib32] Obrand DI, Gordon PH (1997) Incidence and patterns of recurrence following curative resection for colorectal carcinoma. Dis Colon Rectum 40: 15–24 9102255 10.1007/BF02055676

[bib33] Parkin DM, Bray F, Ferlay J, Pisani P (2005) Global cancer statistics, 2002. CA Cancer J Clin 55: 74–108 15761078 10.3322/canjclin.55.2.74

[bib34] Pelosi G, Bresaola E, Bogina G, Pasini F, Rodella S, Castelli P, Iacono C, Serio G, Zamboni G (1996) Endocrine tumors of the pancreas: Ki-67 immunoreactivity on paraffin sections is an independent predictor for malignancy: a comparative study with proliferating-cell nuclear antigen and progesterone receptor protein immunostaining, mitotic index, and other clinicopathologic variables. Hum Pathol 27: 1124–1134 8912819 10.1016/s0046-8177(96)90303-2

[bib35] Pelosi G, Pasini F, Fraggetta F, Pastorino U, Iannucci A, Maisonneuve P, Arrigoni G, De MG, Bresaola E, Viale G (2003a) Independent value of fascin immunoreactivity for predicting lymph node metastases in typical and atypical pulmonary carcinoids. Lung Cancer 42: 203–213 14568688 10.1016/s0169-5002(03)00294-0

[bib36] Pelosi G, Pastorino U, Pasini F, Maissoneuve P, Fraggetta F, Iannucci A, Sonzogni A, De MG, Terzi A, Durante E, Bresaola E, Pezzella F, Viale G (2003b) Independent prognostic value of fascin immunoreactivity in stage I nonsmall cell lung cancer. Br J Cancer 88: 537–547 12592367 10.1038/sj.bjc.6600731 PMC2377175

[bib37] Rodel C, Sauer R (2004) Radiotherapy and concurrent radiochemotherapy for rectal cancer. Surg Oncol 13: 93–101 15572091 10.1016/j.suronc.2004.08.012

[bib38] Sadahiro S, Suzuki T, Ishikawa K, Nakamura T, Tanaka Y, Masuda T, Mukoyama S, Yasuda S, Tajima T, Makuuchi H, Murayama C (2003) Recurrence patterns after curative resection of colorectal cancer in patients followed for a minimum of ten years. Hepatogastroenterology 50: 1362–1366 14571738

[bib39] Tao YS, Edwards RA, Tubb B, Wang S, Bryan J, McCrea PD (1996) beta-Catenin associates with the actin-bundling protein fascin in a noncadherin complex. J Cell Biol 134: 1271–1281 8794867 10.1083/jcb.134.5.1271 PMC2120989

[bib40] Tilney LG, Connelly PS, Vranich KA, Shaw MK, Guild GM (1998) Why are two different cross-linkers necessary for actin bundle formation *in vivo* and what does each cross-link contribute? J Cell Biol 143: 121–133 9763425 10.1083/jcb.143.1.121 PMC2132811

[bib41] Tong GX, Yee H, Chiriboga L, Hernandez O, Waisman J (2005) Fascin-1 expression in papillary and invasive urothelial carcinomas of the urinary bladder. Hum Pathol 36: 741–746 16084942 10.1016/j.humpath.2005.05.005

[bib42] Wong V, Ching D, McCrea PD, Firestone GL (1999) Glucocorticoid down-regulation of fascin protein expression is required for the steroid-induced formation of tight junctions and cell-cell interactions in rat mammary epithelial tumor cells. J Biol Chem 274: 5443–5453 10026156 10.1074/jbc.274.9.5443

[bib43] Xie JJ, Xu LY, Zhang HH, Cai WJ, Mai RQ, Xie YM, Yang ZM, Niu YD, Shen ZY, Li EM (2005) Role of fascin in the proliferation and invasiveness of esophageal carcinoma cells. Biochem Biophys Res Commun 337: 355–362 16185662 10.1016/j.bbrc.2005.09.055

[bib44] Yamashiro S, Yamakita Y, Ono S, Matsumura F (1998) Fascin, an actin-bundling protein, induces membrane protrusions and increases cell motility of epithelial cells. Mol Biol Cell 9: 993–1006 9571235 10.1091/mbc.9.5.993 PMC25324

[bib45] Yamashiro-Matsumura S, Matsumura F (1986) Intracellular localization of the 55-kD actin-bundling protein in cultured cells: spatial relationships with actin, alpha-actinin, tropomyosin, and fimbrin. J Cell Biol 103: 631–640 3525578 10.1083/jcb.103.2.631 PMC2113825

[bib46] Yoder BJ, Tso E, Skacel M, Pettay J, Tarr S, Budd T, Tubbs RR, Adams JC, Hicks DG (2005) The expression of fascin, an actin-bundling motility protein, correlates with hormone receptor-negative breast cancer and a more aggressive clinical course. Clin Cancer Res 11: 186–192 15671545

[bib47] Zhang H, Xu L, Xiao D, Xie J, Zeng H, Cai W, Niu Y, Yang Z, Shen Z, Li E (2006) Fascin is a potential biomarker for early-stage esophageal squamous cell carcinoma. J Clin Pathol 59: 958–964 16524962 10.1136/jcp.2005.032730 PMC1860492

